# The Postural Stability Measures Most Related to Aging, Physical Performance, and Cognitive Function in Healthy Adults

**DOI:** 10.1155/2020/5301534

**Published:** 2020-08-22

**Authors:** Saud F. Alsubaie

**Affiliations:** Department of Physical Therapy and Health Rehabilitation, Prince Sattam Bin Abdulaziz University, Alkharj 16278, Saudi Arabia

## Abstract

**Background:**

Different measures have been used to quantify body balance; some of which use technology to measure postural sway, others are physical performance or self-reported. However, there is little information on the best postural sway measures associated with aging, physical performance, and cognitive function measures.

**Objective:**

To evaluate the relationship between postural sway measures and aging, physical performance, and cognitive function measures.

**Methods:**

A total of 51 subjects (53% female, mean age 53.2 ± 21 years) participated in this cross-sectional study. The participants completed the Activities-specific Balance Confidence (ABC) Scale questionnaire, the Functional Gait Assessment (FGA), the Montreal Cognitive Assessment (MoCA) test, and gait speed. Afterward, the participants performed 8 balance exercises, and their postural sway was measured using a force plate. Spearman's rank correlation coefficient was used to examine the relationship between the study variables.

**Results:**

Age was negatively associated with cognitive function, gait speed, ABC scores, and FGA scores. In addition, cognitive ability was associated positively with ABC scores (*r* = 0.38, *p* ≤ 0.01). Age, FGA scores, and gait speed were significantly associated with the postural sway of the AP direction in some exercises and in all exercises in the ML directions (*p* < 0.05). The cognitive function and ABC scores were significantly associated with only postural sway measures in the ML direction (*p* < 0.05).

**Conclusion:**

The postural sway measures in the lateral direction had more and stronger associations with age, physical performance, and cognitive function measures compared to those in the AP direction.

## 1. Introduction

Control of body balance depends on several factors including the integrity of the sensory organs that provide sensory inputs to the brain about balance, including vision, vestibular apparatus, and somatosensory, as well as the proper integration between these nervous systems. In addition, the ability to respond properly by postural muscles to balance threats is one of the important factors in maintaining balance.

The amount of body sway is considered an appropriate indicator that reflects the ability to maintain balance. A small amount of postural sway reflects better control of body balance, as it requires less effort to maintain balance, while on contrary, increased postural sway indicates a worse balance, which may lead to a fall accident [1, 2].

Several methods have been proposed to measure balance such as using a force plate to quantify the trajectory of the center of pressure which is the resultant of ground reaction force [3, 4] and assumed to proportionally represent the center of mass [3, 5]. Using an accelerometer that attaches to the lower back is considered another way to measure the amount of equilibrium since it is assumed to be closer to the location of the center of mass [5–8]. In addition, a self-rating scale has been validated recently to assess the perceived difficulty of balance exercises [6]. The use of a force plate is still considered the gold standard for measuring the amount of body sway and is widely available and used in clinical settings and research centers. Several studies reported that recording of the amount of body sway using a force plate can predict the possibility of falling [2, 9].

Numerous studies indicated that postural stability, physical activity, and cognitive ability decrease with increasing age [7, 10–15]. However, there is little information related to the relationship between postural stability measures, physical performance, and cognitive ability. Understanding the relationship between balance measures and the level of physical and cognitive activity helps health practitioners in assessing and rehabilitating those who suffer from a deficit in one of these aspects, because in the event that there is a significant relationship among them, it gives an indication that the presence of a problem in one of these aspects may mean that there is a defect in the other aspects, and using a treatment method to improve one of them may improve the other aspects.

This study is aimed at examining the relationship between postural sway measures recorded by a force plate and aging, physical performance, and cognitive function, thus determining which postural sway measure is more related to aging, physical, and cognitive measures in healthy adults.

## 2. Methods

### 2.1. Participants

This is a cross-sectional study that included healthy adults between the ages of 20 and 85 years. The included subjects were able to walk independently without the need for an assistant. Participants with neurological or orthopedic disorders that may affect their balance were excluded. In addition, participants with a cognitive problem who could not understand the assessor's instructions or who were unable to stand for 3 minutes without rest were also excluded. The study was clearly explained to all participants, and then, participants signed a consent form before the start of the experiment. This study was approved by the Institutional Review Board at the University of Pittsburgh.

### 2.2. Experimental Design and Outcome Measures

Eligible participants completed self-report and performance measures at the beginning of their visit including the Activities-specific Balance Confidence (ABC) Scale questionnaire, the Functional Gait Assessment (FGA), the Montreal Cognitive Assessment (MoCA) test, and gait speed. Afterward, participants performed 8 balance exercises, and their postural sway was recorded.

### 2.3. A Self-Reported Measure of Balance Confidence

The Activities-specific Balance Confidence (ABC) Scale questionnaire was used [16]. The purpose of the questionnaire is to record the subject's confidence level in participating in 16 daily activities without losing their balance or feeling unstable. This questionnaire is a self-report measure with scores ranging from 0 to 100, where 0 indicates that the subject does not feel confident whereas 100 indicates that the subject is highly confident.

### 2.4. Functional Performance Measures

Participants performed the Functional Gait Assessment (FGA) test to assess their postural control during 10 walking tasks [17]. The FGA is a 10-item physical test with a score ranging from 0 to 30, where 0 indicates the worse postural control whereas 30 indicates the best postural control.

All subjects' gait speed was measured over 6 meters [18]. Subjects were instructed to walk at their comfortable speed for 10 meters; their speed during the middle 6 meters was measured to avoid the effect of acceleration and deceleration. The data was collected over three trials, and the average of the three trials was calculated.

### 2.5. Cognitive Function Measure

The cognitive ability of the participants was assessed using the Montreal Cognitive Assessment (MoCA) test [19]. The MoCA test is designed to assess different domains of cognition such as memory, orientation, and calculations. The test was administered by an examiner and took about 10 minutes to complete the test on average. The MoCA test's range of score is between 0 and 30; the score of 25 or less is considered a mild cognitive disorder [19].

### 2.6. Postural Sway Measures

Participants performed eight static standing exercises in a random order for each participant and set. Participants were asked to stand as still as possible for 35 seconds for each exercise and took a one-minute seated rest break after every three exercises to minimize the effect of fatigue. Participants were asked also to keep their hands at their side and stand without shoes to minimize the effect of wearing different types of shoes. The feet were kept in the same place during all exercises using a tracing paper, and participants were secured by a safety harness to prevent falling. The included exercises are as follows:

- Standing on a firm surface with eyes open and feet apart stance
- Standing on a firm surface with eyes open and semitandem stance
- Standing on a firm surface with eyes closed and feet apart stance
- Standing on a firm surface with eyes closed and semitandem stance
- Standing on a foam surface with eyes open and feet apart stance
- Standing on a foam surface with eyes open and semitandem stance
- Standing on a foam surface with eyes closed and feet apart stance
- Standing on a foam surface with eyes closed and semitandem stance

Postural sway was measured while participants maintain their balance using a force plate to measure the Root Mean Square (RMS) of linear displacement of the center of pressure (COP) in anteroposterior (AP) and mediolateral (ML) directions. The sway data was measured at a sampling rate of 100 Hz and filtered at a low pass using the 2^nd^ order Butterworth filter and a cutoff frequency of 3 Hz [20, 21]. Although the data were recorded in each exercise for thirty-five seconds, the first five seconds was deleted, and only the remaining thirty seconds was included in the analysis to avoid potential effects when initially establishing balance.

### 2.7. Sample Size

The number of participants needed to achieve the objective of the study was calculated according to the sample size guidelines for correlation analysis [22, 23]. The sample size calculation was computed to reach a minimum correlation coefficient of 0.4 which represents a moderate correlation. Considering this desired correlation coefficient and in order to achieve a power of 80% and type one error of 0.05, we found that the required number of participants in this study was 47 subjects.

### 2.8. Data Analysis

Demographic data and clinical measurements are summarized in means and standard deviations or percentages forms. The Shapiro–Wilk test was used to test the normality of the distribution of the study variables. Because all variables were not normally distributed, Spearman's rank correlation coefficient was used to examine the relationship among the study variables.

## 3. Results

Table 1 shows the demographic data and clinical characteristics of the participants. A total of sixty-two subjects were contacted for participation, and only 51 met the study criteria. The included participants were between the age of 20 and 85 years and had a mean age of 53.2 ± 21 years. On average, the mean scores of the clinical measures are within normal ranges.

### 3.1. Relationships among Age, Physical, and Cognitive Measurements

Age was negatively and significantly (*p* ≤ 0.05) associated with cognitive ability, gait speed, Activities-specific Balance Confidence scores, and Functional Gait Assessment scores. The Activities-specific Balance Confidence scores and gait speed were positively associated with Functional Gait Assessment scores (*r* = 0.531 and 0.529, *p* ≤ 0.01). In addition, the cognitive ability was associated positively with Activities-specific Balance Confidence scores (*r* = 0.38, *p* ≤ 0.01) (Table 2).

### 3.2. Relationships between Postural Sway Measures and Age, Functional Performance, Self-Reported Balance Confidence, and Cognitive Function Measurements

Age was positively and significantly correlated with the RMS of COP displacement in the AP direction in most of the exercises (*r* = 0.34–0.57, *p* ≤ 0.05) and all exercises for the ML direction (*r* = 0.31–0.71, *p* ≤ 0.05) indicating an increase (worsening) in the postural instability with age (Table 3).

The cognitive function was significantly associated with the lateral postural sway measures in one exercise (feet apart eyes closed foam surface) only, (*r* = −0.33, *p* ≤ 0.05). However, cognitive function was not significantly associated with postural sway measures of the AP direction in all exercises. Similarly, the ABC questionnaire results were not significantly associated with all postural sway measures of the AP direction but were associated with the lateral postural sway measures in some exercises, with correlation coefficients ranging from -0.42 to -0.30 (*p* ≤ 0.05) (Table 3).

Functional measures (FGA and gait speed) were negatively associated with the postural sway measures in more exercises in the ML direction (seven and four exercises, respectively) compared to the AP direction (four and two exercises, respectively) (Table 3).

## 4. Discussion

In this study, we examined the relationship between postural sway measures that were collected through a force plate with physical performance, cognitive ability, and a perceptual measure of how confident participants were not to fall while performing some daily life activities.

On average, the mean scores of the clinical measures including cognitive ability, gait speed, FGA scores, and participants' confidence that they can perform some daily life activities without losing their balance were within normal ranges [16, 24–30].

We found in this study that the greater the age, the less cognitive ability, walking speed, scores of the FGA, and participants' perception of their confidence not to fall while performing daily life tasks. These results were not surprising, and a large number of research studies indicated such results [7, 15].

Most participants achieved high scores on the cognitive ability scale (MoCA), and this may have resulted in a low number of relationships between its scores and physical measures (FGA and gait speed); despite that there was a positive relationship between cognitive ability and the participants' perception of their confidence in performing some daily life tasks without losing their balance. In a study that included adolescents with sports concussion, they found that the perceptual measure ABC was more closely related to more subcategories of the cognitive ability scale compared to physical measures such as walking speed and Functional Gait Assessment [31]. The reason behind this relationship between the cognitive ability and the participants' confidence in performing some daily tasks without losing their balance may be due to the fact that both measures depend on the perception of the participant himself, unlike other measures that depend on physical performance.

The amount of postural sway (RMS of the COP displacement) in the AP and ML directions increased (worsen) with increasing age, and this result is consistent with the results of several studies [10–15]. Age was associated with the RMS of the COP displacement in the ML direction in all exercises, while age was associated with the RMS of the COP displacement in the AP direction in some exercises, especially those with semitandem stance. Previous studies have shown that postural sway measures with the most challenging foot positions are more sensitive to differences between age groups [15, 32]. This indicates that the postural sway measures in the AP direction may need more challenging exercises to be sensitive to age differences, whereas the lateral postural sway measures were related to the age increase in all exercises regardless of the varying difficulty.

FGA scores and gait speed were negatively related to the RMS of the COP displacement in the AP direction in few exercises and were associated with the RMS of the COP displacement in the ML direction in more exercises. Furthermore, the cognitive function and participants' ratings of their confidence in their balance correlated with postural sway measures in the ML direction only. Several studies indicated that lateral postural sway measures were more sensitive to age differences, different pathological groups, and the differences between balance tasks with varying difficulty levels [14, 15, 32, 33] and can predict future falls [34–36]. Moreover, a recent study found that lateral postural sway measures were more closely related to participants' perceptual assessment of exercise difficulty compared to sagittal postural sway measures [6]. Several studies also indicated that the magnitude of postural sway in the ML direction has more reliability [7, 37–42] and was correlated with more physical [42] and self-reported measures [6] as well as number of future falls [34–36] compared to that in the AP direction. Several studies provided an explanation for that, where they indicated that the lateral sway has more variation due to the narrow support base while performing balance exercises compared to the sagittal sway, which may allow for greater reliability and more relationships with different measures [6, 7]. Therefore, it is worthwhile to use the lateral postural sway measures to quantify balance performance and to monitor the improvement in the stability of participants in balance rehabilitation programs.

The Functional Gait Assessment questionnaire was related to most of the measures used in the study including postural sway in both the AP and ML directions. The FGA questionnaire has been developed by modifying a previous functional scale (Dynamic Gait Index) [17] and has been validated in different age and pathological populations such as Parkinson's disease, stroke, and vestibular disorders [17, 43–45]. The relationship of the Functional Gait Assessment questionnaire with most of the measures included in the study indicates that it is an important tool to be used in rehabilitation clinics as it gives indications of the physical ability and the extent of a person's confidence in his ability to perform some daily life tasks without losing their balance as well as the postural stability. Several studies also indicated the importance of the Functional Gait Assessment questionnaire, as it was found to be related to several physical, balance, and cognitive measures as well as to the risk of falling [17, 43, 46].

The negative relationship between the postural sway in the ML direction and the ABC scale indicates that participants with more sway had less confidence in their ability to maintain their balance while performing several daily activities. Several studies also found similar results despite using various measures to quantify subjects' confidence in their balance when compared to the amount of postural sway [42, 47].

## 5. Conclusion

The postural sway in the ML direction was associated with all study variables including age, physical, and cognitive measures. Therefore, it is worthwhile to use the lateral postural sway measures for functional assessment and to guide the clinical decision. Similarly, the FGA test was related to various measures including age, gait speed, and perceptual measure of participants' level of confidence in maintaining their balance without falling while performing some daily life activities, in addition to its association with measures of the postural stability. The availability of FGA and the ease of use in clinic allow health practitioners to assess physical performance and obtain an impression of other medical aspects of their clients.

## Figures and Tables

**Table 1:** Demographic and clinical characteristics of the participants (*n* = 51).

Variable	Mean ± SD or %
Age (years)	53.2 ± 21
Gender (female/male)	27/24
Body mass index (BMI) (kg/m^2^)	26.2 ± 5
Montreal Cognitive Assessment	28.6 ± 1
Activities-specific Balance Confidence Scale questionnaire	95.6 ± 4
Gait speed	1.31 ± 0.2
Functional Gait Assessment	27.4 ± 3

**Table 2:** Correlation coefficients of the relationships among clinical and physical measurements.

	Age	BMI	MoCA	ABC	Gait speed
BMI	0.408^∗∗^				
MoCA	-0.294^∗^	-0.243			
ABC	-0.338^∗^	-0.211	0.381^∗∗^		
Gait speed	-0.400^∗∗^	-0.197	-0.052	0.198	
FGA	-0.696^∗∗^	-0.298^∗^	0.196	0.531^∗∗^	0.529^∗∗^

∗Significance level ≤ 0.05, ∗∗significance level ≤ 0.01.

**Table 3:** Correlation coefficients of the relationships between postural sway measures and study variables.

	Age	MoCA	ABC	Gait speed	FGA
Displacement of COP (AP)					
Feet apart eyes open firm surface	0.222	0.141	-0.020	-0.242	-0.298
Semitandem eyes open firm surface	0.417∗∗	0.002	-0.085	-0.176	-0.296∗
Feet apart eyes closed firm surface	0.113	0.027	-0.130	-0.100	-0.097
Semitandem eyes closed firm surface	0.338∗	-0.186	-0.116	0.006	-0.082
Feet apart eyes open foam surface	0.371∗∗	0.035	-0.063	-0.403∗∗	-0.390∗∗
Semitandem eyes open foam surface	0.531∗∗	-0.151	-0.225	-0.313∗	-0.428∗∗
Feet apart eyes closed foam surface	0.159	-0.202	-0.131	-0.181	-0.070
Semitandem eyes closed foam surface	0.526∗∗	-0.122	-0.087	-0.167	-0.154
Displacement of COP (ML)					
Feet apart eyes open firm surface	0.308∗	0.146	-0.026	-0.142	-0.261
Semitandem eyes open firm surface	0.525∗∗	-0.004	-0.040	-0.350∗	-0.535∗∗
Feet apart eyes closed firm surface	0.401∗∗	-0.074	-0.223	-0.116	-0.312∗
Semitandem eyes closed firm surface	0.498∗∗	-0.247	-0.302∗	-0.020	-0.314∗
Feet apart eyes open foam surface	0.566∗∗	-0.133	-0.334∗	-0.309∗	-0.572∗∗
Semitandem eyes open foam surface	0.686∗∗	-0.143	-0.196	-0.373∗∗	-0.513∗∗
Feet apart eyes closed foam surface	0.707∗∗	-0.329∗	-0.419∗∗	-0.312∗	-0.535∗∗
Semitandem eyes closed foam surface	0.663∗∗	-0.135	-0.214	-0.221	-0.423∗∗

∗Significance level ≤ 0.05, ∗∗significance level ≤ 0.01.

## Data Availability

The data is available upon reasonable request.
